# CAREGIVING CULTURES: RACE, REGION, AND RELATIONS

**DOI:** 10.1093/geroni/igz038.667

**Published:** 2019-11-08

**Authors:** Jyoti Savla, Karen A Roberto, Rosemary Blieszner

## Abstract

Although families have always cared for their aging members, changes in contemporary society have added to the stress and challenges of providing daily care and support for relatives experiencing physical and/or cognitive decline. Personal characteristics, geographic location, and family structures and relationships influence beliefs about family care; thus, recognizing differences within and across families is crucial for developing culturally informed caregiving programs and practices. This symposium focuses on four diverse groups of caregivers. Recognition of caregiving cultures and the barriers caregivers encounter is the central theme of all four papers. Using daily diary interviews and GIS data, Savla and colleagues discuss the unique cultural and geographical challenges caregivers in rural Appalachian Virginia face when caring for a family member with dementia. J. Angel employs quantitative and qualitative data from two nationally representative datasets to discuss the effects of immigration on family structures and caregiving for Mexican-American older adults with dementia. Miyawaki and her colleagues provide a profile of caregivers of older Vietnamese refugees, the resources they use and support structures they rely on. Finally, Roberto and Savla expand the definition of family caregivers to include extended and fictive kin who are providing dementia care and provide an in-depth view of the circumstances that influence the responsibilities they assumed, the type of care they provide, and the coping strategies they use. Dr. Rosemary Blieszner will discuss the presenters’ collective findings considering their unique caregiving practices and beliefs as well as the common grounds between the different races, regions and relationships.

